# The Evolving Role of Radiotherapy for Pediatric Cancers With Advancements in Molecular Tumor Characterization and Targeted Therapies

**DOI:** 10.3389/fonc.2021.679701

**Published:** 2021-09-16

**Authors:** Colette J. Shen, Stephanie A. Terezakis

**Affiliations:** ^1^Department of Radiation Oncology, University of North Carolina, Chapel Hill, NC, United States; ^2^Department of Radiation Oncology, University of Minnesota, Minneapolis, MN, United States

**Keywords:** precision medicine & genomics, pediatric cancer, targeted therapies, molecular diagnostics, radiation therapy (radiotherapy), pediatric glioma, medulloblastoma, pediatric sarcomas

## Abstract

Ongoing rapid advances in molecular diagnostics, precision imaging, and development of targeted therapies have resulted in a constantly evolving landscape for treatment of pediatric cancers. Radiotherapy remains a critical element of the therapeutic toolbox, and its role in the era of precision medicine continues to adapt and undergo re-evaluation. Here, we review emerging strategies for combining radiotherapy with novel targeted systemic therapies (for example, for pediatric gliomas or soft tissue sarcomas), modifying use or intensity of radiotherapy when appropriate *via* molecular diagnostics that allow better characterization and individualization of each patient’s treatments (for example, de-intensification of radiotherapy in WNT subgroup medulloblastoma), as well as exploring more effective targeted systemic therapies that may allow omission or delay of radiotherapy. Many of these strategies are still under investigation but highlight the importance of continued pre-clinical and clinical studies evaluating the role of radiotherapy in this era of precision oncology.

## Introduction

In the early history of pediatric cancer treatment, surgical resection and then radiation therapy served as the primary treatment modalities (1, 2). Subsequent introduction of chemotherapy regimens resulted in combination therapies with reduction in radiotherapy dose in many cases (3, 4). Further refinement of chemotherapy regimens and significant advancements in radiotherapy techniques have led to improvements in disease outcomes while limiting late toxicities, critical for treatment of childhood cancers. Recently, dramatic and rapid advancements in precision medicine, which we define here as more precise genomic and molecular characterization of individual tumors, development of targeted anti-tumor drugs, and improved accuracy and conformality of radiotherapy, have enabled treatment approaches that may be better tailored to each patient (5–8). Radiotherapy has remained a mainstay and one of the most effective anti-cancer treatments; however, these advances in precision medicine require constant re-evaluation of the role of radiotherapy in this evolving landscape. A critical goal in the treatment of pediatric malignancies is to maintain effective cancer control while minimizing late toxicities as much as possible. On one hand, it can be tempting to try to omit or limit the use of radiotherapy for childhood cancers given potential late effects in an era of improvements in targeted systemic therapies. In some cases, this may be appropriate for select patients, as long as disease control can be maintained. On the other hand, the potential for radiotherapy to synergize with targeted drugs should be explored and fully utilized. Significant advancements in radiotherapy techniques have also been made in this era of precision medicine, *via* improvements in conformality with intensity-modulated radiotherapy (IMRT) and proton therapy, better precision with image guidance, and reductions of dose and treatment volumes where appropriate, allowing for reduced toxicity and an improved therapeutic ratio with radiotherapy.

## Role of Radiotherapy With Advances in Targeted Systemic Therapies

Better molecular and genomic characterization of tumors, along with advances in targeted drug development, have resulted in more specific systemic therapies for pediatric tumors, which in some cases may have better anti-tumor efficacy and in many cases are associated with less toxicity compared to standard chemotherapy regimens. In some cases, these targeted systemic therapies can be used upfront, delaying local radiotherapy and reserving it for progression, while in others, these targeted therapies may be given concurrently with or following radiotherapy, or in the recurrent or metastatic setting.

### Management of Pediatric Low-Grade Gliomas With Advances in Targeted Therapies

**Figure 1:**
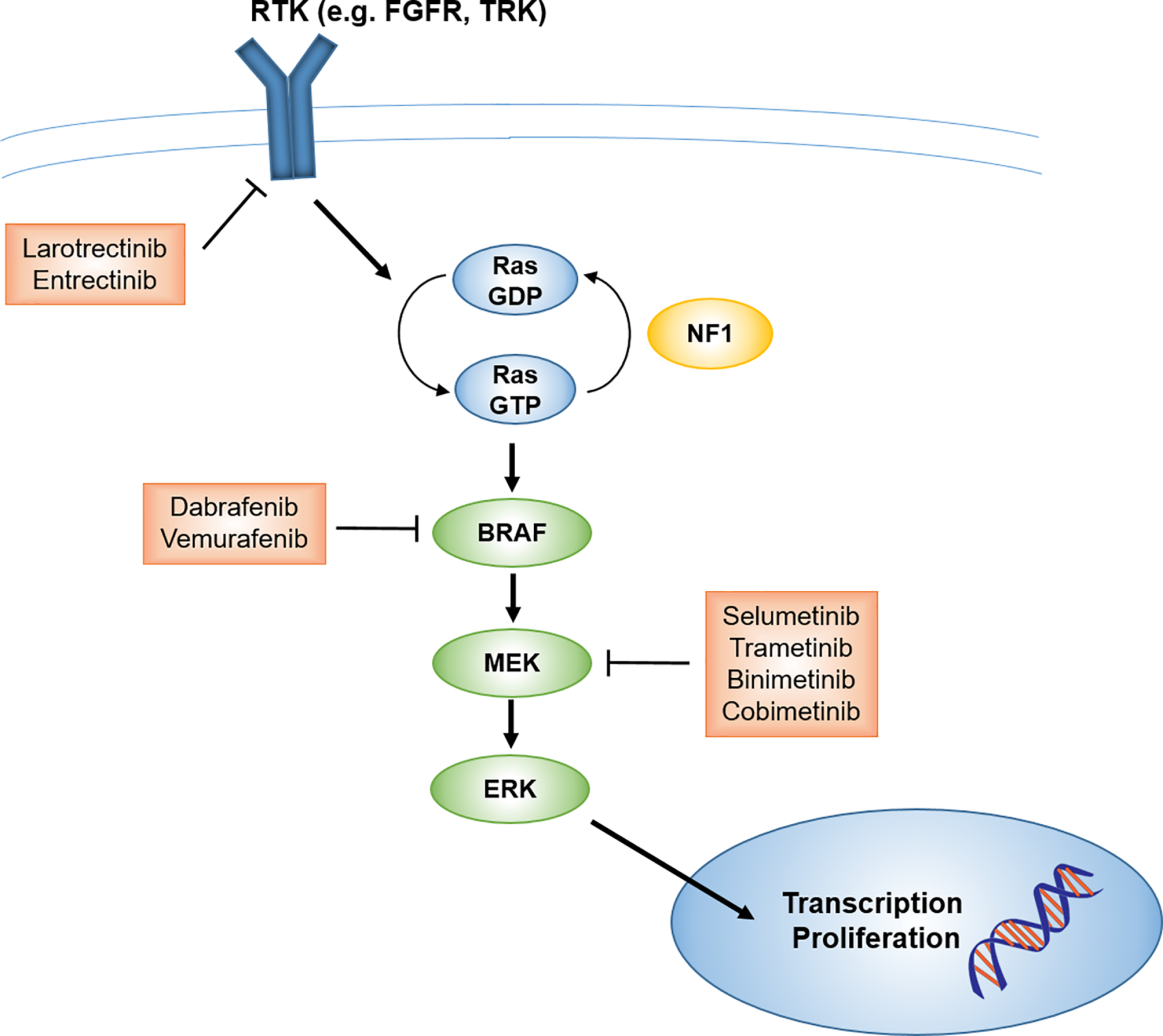
Schematic of MAPK signaling pathway and potential targets and therapeutics for pediatric LGG. FGFR, fibroblast growth factor receptor; LGG, low-grade glioma; MAPK, mitogen-activated protein kinase; RTK, receptor tyrosine kinase; TRK, tropomyosin receptor kinase.

**Figure 2:**
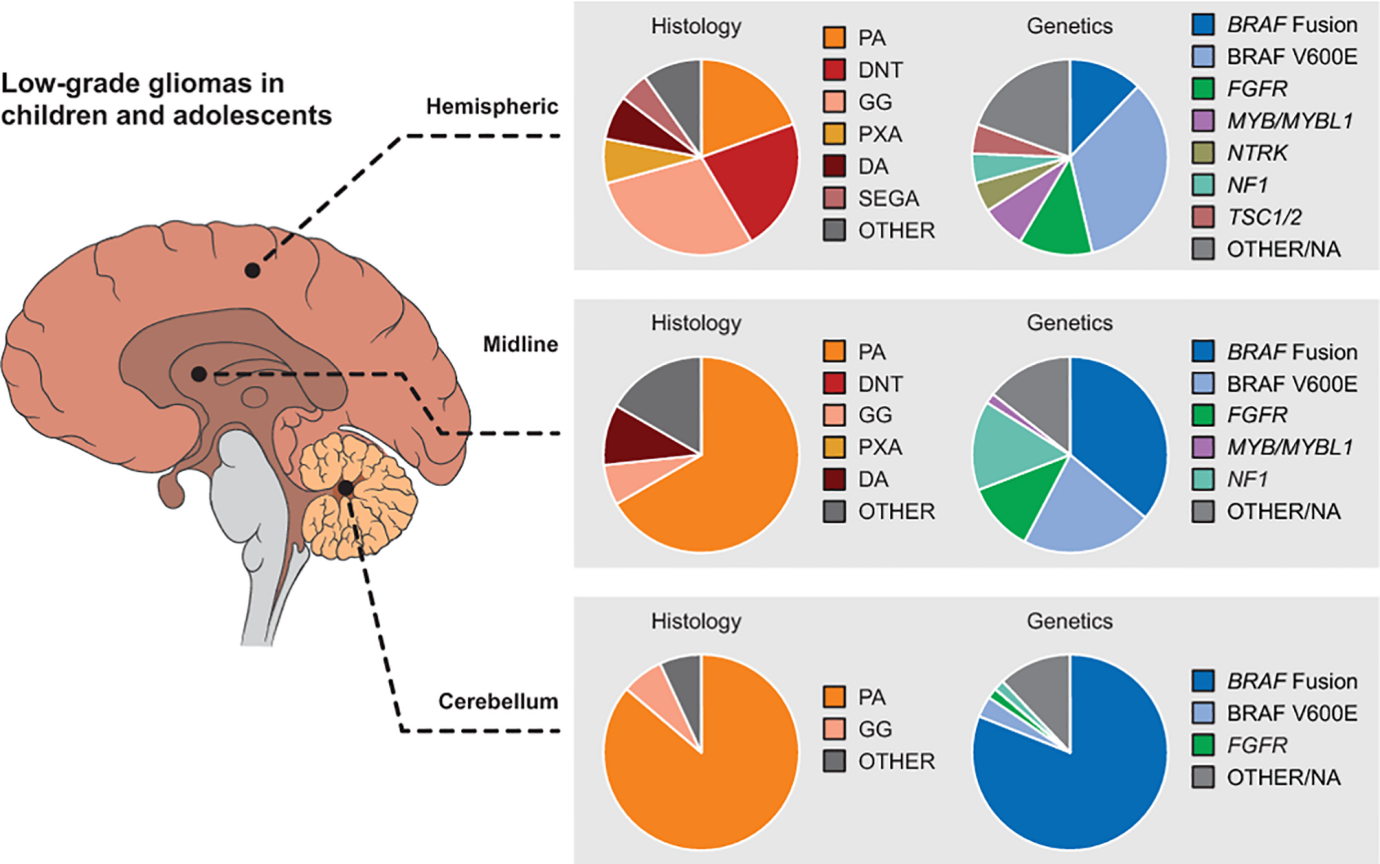
Distribution of pediatric LGG histologies and genetic alterations by location in the brain. Reproduced with permission from Filbin and Sturm (12). DA, diffuse astrocytoma; DNT, dysembryoplastic neuroepithelial tumors; GG, ganglioglioma; LGG, low-grade glioma; PA, pilocytic astrocytoma; PXA, pleomorphic xanthoastrocytomas; SEGA, subependymal giant cell astrocytoma.

Low-grade gliomas (LGG) are among the pediatric tumor types for which novel targeted agents have demonstrated promising potential. While malignant progression is rare in pediatric LGG (in contrast to adult LGG) and 5-year overall survival is greater than 90% (9), patients whose tumors cannot be fully resected often end up requiring multiple courses of therapy, with associated late effects and long-term reduction in quality of life (10). For LGG that cannot be managed by surgery alone, current management is controversial: conventional cytotoxic chemotherapy is typically the recommended initial approach for pediatric patients, deferring radiotherapy to limit late toxicities (11). However, advances in radiotherapy techniques that can reduce late toxicities, including IMRT and proton therapy, may make radiotherapy a more viable earlier-line option. Further, it is now fairly established that the majority of pediatric LGG arise from an alteration in the mitogen-activated protein kinase (MAPK) signaling pathway, including BRAF mutation (most commonly V600E point mutation) or fusion (most commonly BRAF : KIAA1549), NF1 mutation, NTRK family fusion, and FGFR1 mutation or rearrangement, along with other less common alterations (**Figures 1**, **2**) (5, 6, 13–16). Thus, targeted agents including MEK1/2 (an upstream kinase of MAPK), BRAF, and TRK inhibitors have been evaluated and have demonstrated promising activity in pediatric gliomas (17–22).

The most mature data in this setting exist for the MEK1/2 inhibitor selumetinib. In a multicenter phase 2 study by the Pediatric Brain Tumor Consortium, pediatric patients with recurrent, refractory, or progressive LGG after at least one line of standard therapy were treated with selumetinib (18). Response and survival outcomes compare favorably to prior studies of recurrent or progressive pediatric LGG treated with chemotherapy regimens including carboplatin/vincristine and vinblastine monotherapy (**Table 1**) (18, 23–27). We note that data regarding the efficacy of selumetinib for patients without NF1- or BRAF alteration-associated LGG from this study are still pending, and prior studies of chemotherapy did not stratify or have information regarding NF1 or BRAF status. Nonetheless, these promising results have led to the current Children’s Oncology Group (COG) randomized studies ACNS1831 [NCT03871257] and ACNS1833 [NCT04166409], which are evaluating selumetinib *versus* standard carboplatin/vincristine chemotherapy in the upfront setting for patients with NF1-associated or non-NF1-associated low grade gliomas, respectively.

**Table 1:** Prospective studies of systemic therapies for recurrent/progressive/refractory pediatric low-grade glioma.

Study (Accrual years)	Number of patients	Patient population	Study type	Systemic therapy agent(s)	ORR	EFS/PFS	OS
Packer et al. (23)(accrual years not reported)	N=23 (recurrent) N=37 (newly diagnosed)	Age <21 years with recurrent LGG or age <5 years with progressive, newly diagnosed LGG	Single arm (multi-center)	CARBO/VCR	Recurrent: 52 ± 10% Newly diagnosed: 62 ± 8%	NR	NR
Gururangan et al. (24)(1993-2000)	N=81	Age ≤18 years with progressive LGG	Single arm phase 2 (multi-center)	CARBO	28% (95% CI 18-38%)	3-year FFS: 64% (95% CI 54-76%)	3-year OS: 84% (95% CI 76-93%)
Ater et al. (25)(1997-2005)	N=274	Age <10 years with progressive or residual LGG	Randomized (COG)	CARBO/VCR *vs*. TPCV	CARBO/VCR: 50% TPCV: 52%	5-year EFS (all patients): 45 ± 3% (difference between arms NS)	5-year OS (all patients): 86 ± 2%
Bouffet et al. (26)(2002-2006)	N=51	Age <21 years with recurrent or refractory LGG	Single arm phase 2 (multi-center)	Vinblastine	36%	5-year EFS: 42 ± 7%	5-year OS: 93 ± 4%
Fangusaro et al. (18)(2013-2015)	N=25 (Stratum 1: pilocytic astrocytoma with BRAF aberration) N=25 (Stratum 3: NF1-associated LGG)	Age 3-21 years with recurrent, refractory, or progressive LGG (≥1 prior line of therapy)	Single arm phase 2 (PBTC)	Selumetinib	Stratum 1: 36% Stratum 3: 40%	2-year PFS: Stratum 1: 70% (95% CI 47-85%) Stratum 3: 96% (95% CI 74−99%	NR
Hargrave et al. (19)(2013-2015)	N=32	Age <18 years with BRAF V600-mutant recurrent, refractory, or progressive LGG (≥1 prior line of therapy)	Single arm phase 1/2a (multi-center)	Dabrafenib	44% (95% CI 26-62%)	1-year PFS: 85% (95% CI 64-94%)	NR

COG, Children’s Oncology Group; CARBO, carboplatin; CI, confidence interval; EFS, event-free survival; FFS, failure-free survival; LGG, low-grade glioma; NR, not reported; NS, non-significant; ORR, objective response rate; OS, overall survival; PFS, progression-free survival; PBTC, Pediatric Brain Tumor Consortium; TPCV, thioguanine, procarbazine, lomustine (CCNU), vincristine; VCR, vincristine.

Studies of other targeted agents are also complete or underway, including a phase 2 study (TRAM-01, NCT03363217) of the MEK1/2 inhibitor trametinib (the first FDA-approved MEK inhibitor) in patients with progressing/refractory LGG or plexiform neurofibroma with activation of the MAPK pathway (28), and a phase 1/2 study of the BRAF V600 inhibitor dabrafenib in pediatric patients with BRAF V600-mutant relapsed or refractory LGG (**Table 1**) (19, 29). BRAF V600E mutation has been identified in nearly 20% of pediatric LGG across a range of histologies and sites and confers a worse prognosis than BRAF wild-type tumors when treated with conventional adjuvant therapies (including chemotherapy and radiotherapy) (15). While TRK fusions are less commonly identified in pediatric gliomas, robust responses to TRK kinase inhibitors have been seen in pediatric solid tumors harboring TRK fusions, including high grade gliomas (20, 21, 30). Thus, when feasible, pediatric LGG should be evaluated for potentially targetable alterations, as MEK1/2, BRAF, and TRK inhibitors have demonstrated promising activity in pediatric gliomas and can be considered for patients who have failed upfront chemotherapy.

The timing of use of radiotherapy for LGG is controversial and continues to evolve with developments in targeted systemic therapies and radiotherapy techniques. Radiotherapy has for years demonstrated effective control of unresectable, progressive LGG, with 10-year PFS and overall survival (OS) of approximately 70% and 80%, respectively (31–33). However, concerns of late toxicity, including neurocognitive deficits, stroke, endocrine dysfunction, and secondary malignancy, especially in younger patients treated with radiotherapy (32–35), led to a shift toward initial treatment with systemic therapy and avoidance or delay of radiotherapy (36–38). In many cases, treatment with multiple lines of systemic therapy, deferring radiotherapy, has resulted in significant morbidity from tumor progression (39). Advances in radiotherapy techniques since the 1990s have allowed for more precise and conformal delivery of radiotherapy, maintaining tumor control while reducing normal tissue toxicity (**Table 2**). An early study of stereotactic radiotherapy for pediatric low-grade gliomas in the 1990s at the Dana Farber Cancer Institute used magnetic resonance imaging (MRI)-based treatment planning and smaller radiotherapy target margins and demonstrated maintained PFS and OS (65% and 82%, respectively, at 8 years), with no marginal failures (40). A subsequent phase 2 trial was conducted at the St. Jude Children’s Research Hospital of conformal radiotherapy for pediatric low-grade gliomas using primarily 3-dimensional conformal radiotherapy (3D-CRT) with a 10mm clinical target volume (CTV) margin and MRI-based planning. Disease control was similarly maintained, with 10-year EFS and OS of 74% and 96%, respectively (41). Late effects were overall limited compared to patients treated with less conformal techniques, although cognitive deficits and risk of vasculopathy were greater in patients younger than age 5 at the time of treatment (41, 45). More recently, the COG study ACNS0221 (2006–2010) evaluated conformal radiotherapy for pediatric LGG, using a smaller 5mm CTV margin with the majority (71%) of patients receiving IMRT, the current standard radiotherapy technique. This study also demonstrated favorable disease control (5-year PFS and OS of 71% and 93%, respectively) with limited toxicity (42). Finally, treatment with proton therapy, which can often further spare normal tissues for pediatric brain tumors compared to IMRT (46), has demonstrated reduced toxicity while maintaining excellent disease control for pediatric LGG. A study from the Massachusetts General Hospital demonstrated 8-year PFS and OS of 83% and 100%, respectively, and no significant declines in intelligence quotient (IQ), although a subset analysis suggested more neurocognitive decline in patients <7 years and those with significant dose to the left temporal lobe/hippocampus (43). More recently, a report on a large series of patients (n=174) treated with proton therapy for LGG at the University of Florida Health Proton Therapy Institute also demonstrated excellent disease control (5-year PFS and OS of 84% and 92%, respectively), with <5% developing serious late toxicity at a median follow-up of 4.4 years (44).

**Table 2:** Studies of advanced radiotherapy techniques for pediatric low-grade glioma.

Study (Treatment years)	Number of patients	Patient population	Radiotherapy dose, technique, and margin	Sites of failure	EFS/PFS	OS
Marcus et al. (40) (1992-1998)	N=50	Age 2-26 years with LGG	Dose: Mean 52.2 Gy (range 50.4-58 Gy) Technique: Stereotactic RT, MRI-based planning CTV margin: 0mm PTV margin: 2mm	Marginal: 0 In-field: 6 Distant: 5	8-year PFS: 65%	8-year OS: 82%
Merchant et al. (41)(1997-2006)	N=78	Age 2-19 years with LGG	Dose: 54 Gy Technique: 3D-CRT, MRI-based planning CTV margin: 10mm PTV margin: 3-5mm	Marginal: 1 In-field: 8 Distant: 4	10-year EFS: 74 ± 15%	10-year OS: 96% ± 6%
Cherlow et al. (42) ACNS0221 (2006-2010)	N=85	Age 3-21 years with unresectable progressive, residual, recurrent LGG	Dose: 54 Gy Technique: IMRT (71%), MRI-based planning CTV margin: 5mm PTV margin: 3-5mm	Marginal: 0 In-field: 19 Distant: 4	5-year PFS: 71% ± 6%	5-year OS: 93% ± 4%
Greenberger et al. (43)(1995-2007)	N=32	Age 2-21 years with LGG	Dose: Median 52.2 GyRBE (range 48.6-54 GyRBE) Technique: Proton RT, MRI-based planning CTV margin: 3-5mm PTV margin: N/A	Marginal vs. in-field (not specified): 4 Distant: 1	8-year PFS: 83%	8-year OS: 100%
Indelicato et al. (44)(2007-2017)	N=174	Age 2-21 years with LGG	Dose: 54 GyRBE (74%) Technique: Proton RT, MRI-based planning CTV margin: 5mm PTV margin: 3mm	Marginal: 0 In-field: 21 Distant: 2 In-field + distant: 1	5-year PFS: 84% (95% CI 77-89%)	5-year OS: 92% (95% CI 85-95%)

3D-CRT, 3-dimensional conformal radiotherapy; CI, confidence interval; CTV, clinical target volume; EFS, event-free survival; Gy, Gray; IMRT, intensity-modulated radiotherapy; LGG, low-grade glioma; MRI, magnetic resonance imaging; OS, overall survival; PFS, progression-free survival; PTV, planning target volume; RBE, relative biological effectiveness; RT, radiotherapy.

In this context of reduced toxicity from newer radiotherapy techniques, recent studies suggest that delayed radiotherapy may be associated with worse outcomes in some patients with pediatric LGG. A study of pediatric patients treated with radiotherapy for optic pathway and hypothalamic LGG at St. Jude found that receipt of chemotherapy prior to radiotherapy was associated with worse EFS (hazard ratio 3.1, 95% CI: 1.4-7.0, P=0.007) and that younger age <6 years at the time of radiotherapy (patients who were typically treated first with chemotherapy) had worse EFS and OS (32). A very recent study by investigators at St. Jude reviewed pediatric patients with unresectable LGG treated with radiotherapy and identified low- and high-risk groups based on OS [10-year OS of 96% (95% CI: 89-98%) *versus* 76% (95% CI: 59-87%) respectively] (47). Within the high-risk group, which included diffuse astrocytoma or location within the thalamus/midbrain, delayed radiotherapy (after at least one line of chemotherapy) was associated with worse PFS (hazard ratio 2.5, 95% CI: 1.4-4.4, P=0.001). Thus, early radiotherapy should be considered for LGG patients with higher risk disease, those at risk of functional impairment with progression, older patients, and those without targetable alterations.

Several questions arise from these studies regarding the management of pediatric LGG: can novel targeted agents be combined with radiotherapy, and can modifications in radiotherapy dose be considered? The studies of MEK1/2 and BRAF inhibitors for pediatric LGG have been for recurrent, refractory, or progressive disease and not in combination (whether concurrent or sequential) with radiotherapy. Pre-clinical data have suggested synergy between MEK1/2 and BRAF inhibitors with radiotherapy for pediatric gliomas (48–50), but concerns regarding toxicity of concurrent treatment exist (51, 52). The standard radiotherapy dose for pediatric LGG (~54 Gy) is largely derived from adult studies, where dose escalation above 45-50 Gy has not been associated with improved outcomes in randomized trials, but retrospective data in both adult and pediatric studies suggest better survival with treatment to ≥53 Gy (44, 53–55). As recent studies of radiotherapy for pediatric LGG have focused on reduced margins and more conformal delivery techniques (reviewed above), the standard dose has remained ~54 Gy. While improvements in conformality may lessen the benefits of dose reduction for LGG, there would likely still be significant benefit for patients with larger tumors or those near critical structures such as the hippocampi (56). Further, combination with MEK1/2 and/or BRAF inhibitors may allow for reduction of radiotherapy dose while maintaining tumor control. Future investigations could evaluate these combinations, with standard *versus* reduced-dose radiotherapy and with targeted therapy and radiotherapy delivered concurrently *versus* sequentially as in ACNS1723 for high-grade glioma (discussed in the next section) to minimize toxicities of combined therapy.

### Management of Pediatric High-Grade Gliomas With Advances in Targeted Therapies

While pediatric high-grade gliomas (HGG) are standardly treated with conventional radiotherapy and temozolomide chemotherapy based on adult data (57), this treatment approach as studied in ACNS0126 and ACNS0423 did not improve outcomes in children with HGG compared to prior treatments with radiotherapy and other chemotherapy regimens (58–60). Pediatric diffuse midline gliomas, including diffuse intrinsic pontine glioma (DIPG), are typically considered high grade given aggressive behavior even with lower grade histology (61) and are treated with radiotherapy and best supportive care. Outcomes overall are still very poor for these tumors, and thus novel treatment approaches are desperately needed. Multiple studies have now established a different molecular genetic profile underlying pediatric HGG compared to adult disease, with frequent somatic mutations in histone H3 genes, TP53, and ATRX; focal amplification of PDGFRA; chromosome 1q gain; NTRK and other targetable gene fusions in infant HGG; and infrequent IDH1 hotspot mutations (14, 21, 62–65). Approximately 5-10% of pediatric HGGs harbor BRAF V600E mutations and have a slightly better clinical outcome, potentially accounting for some of the long-term survivors in pediatric HGG trials (66, 67).

Therapeutically, these advances in molecular characterization will allow tailoring of treatment approaches for pediatric HGG instead of a single standard paradigm for all patients. Unfortunately, in contrast to LGG, a single drug is unlikely to benefit a large number of patients given the heterogeneity of these tumors, and radiotherapy will likely remain a critical component of upfront treatment for these patients. Infant HGG may be one subset where targeted therapies are used upfront, deferring radiotherapy, as these tumors more frequently exhibit targetable MAPK alterations and gene fusions targeting ALK, NTRK, ROS1, and MET (14, 21) and have demonstrated rapid clinical responses to targeted therapies in case reports (20, 68). For older children with HGG, two ongoing COG trials are evaluating novel systemic therapies together with radiotherapy depending on tumor molecular features: for patients with BRAF V600 mutant-HGG, ACNS1723 [NCT03919071] is a phase 2 trial evaluating treatment with the BRAF V600 inhibitor dabrafenib and MEK 1/2 inhibitor trametinib following radiotherapy. For those without BRAF V600 or H3 K27M mutations, ACNS1721 [NCT03581292] is a phase 2 trial evaluating concurrent radiotherapy with the poly (ADP-ribose) polymerase (PARP) inhibitor veliparib, followed by maintenance chemotherapy with veliparib and temozolomide. PARP inhibitors, as DNA damage response inhibitors, can effectively synergize with radiotherapy (69, 70) and have demonstrated radio- and chemo-sensitization in pre-clinical studies of glioblastoma (71). PARP inhibition has been evaluated clinically in combination with temozolomide in recurrent adult glioblastoma and recurrent pediatric brain tumors (72, 73), as well as in combination with radiation and temozolomide in the Pediatric Brain Tumor Consortium (PBTC) study PBTC-033 for newly diagnosed DIPG but did not improve survival compared to historical series (74) (thus patients with H3 K27M mutations are excluded from ACNS1721). Along similar lines, Wee1 is a cell cycle regulator that is also involved in the DNA damage repair pathway. Based on promising pre-clinical data (75), the COG is conducting a phase 1 trial of the Wee1 inhibitor adavosertib with radiotherapy for newly diagnosed DIPG (COG-ADVL1217, NCT01922076).

### Management of Pediatric Sarcomas and Other Extracranial Solid Tumors With Advances in Targeted Therapies

Outside of the central nervous system (CNS), targeted systemic therapies are increasingly incorporated in the treatment of pediatric sarcomas, as well as other tumors based on specific molecular and genetic alterations. These are typically included concurrently with radiotherapy as part of definitive treatment, or following standard of care therapy in the recurrent or refractory setting. Based on clinical efficacy in the treatment of adult soft tissue sarcoma (STS) and renal cell carcinoma, pazopanib, a multikinase angiogenesis inhibitor targeting vascular endothelial growth factor receptors (VEGFR), c-kit, and platelet-derived growth factor receptors (PDGFR), was initially evaluated in a phase 1 trial by the COG for children with STS and other refractory solid tumors. This study demonstrated pazopanib was well tolerated in children, had evidence of anti-angiogenic effect, and had potential clinical benefit in pediatric sarcoma (76). Subsequently, the COG together with the adult cooperative group NRG Oncology conducted a randomized phase 2 trial, ARST1321, evaluating the addition of pazopanib to pre-operative chemoradiotherapy for children and adults with large, unresectable, intermediate- or high-grade STS. Initial results after the second interim analysis have recently been published and demonstrated improvement in the pathological near-complete response rate with addition of pazopanib (≥90% pathological response in 58% of patients in the pazopanib group *versus* 22% of patients in the control group) (77). Longer-term follow-up will be required to compare survival outcomes.

Targeted therapy is also being evaluated for newly diagnosed metastatic Ewing sarcoma. Prior phase 1 and phase 2 studies demonstrated favorable responses to ganitumab, an insulin-like growth factor receptor (IGFR) inhibitor, in patients with relapsed or refractory Ewing sarcoma (78, 79). Based on these data, the COG randomized phase 3 trial AEWS1221 is evaluating addition of ganitumab to standard multi-agent chemotherapy for newly diagnosed metastatic Ewing sarcoma [NCT02306161]. Local control with surgery and/or radiotherapy after induction chemotherapy, as well as metastatic site radiotherapy following consolidation chemotherapy, remain components of treatment on this study.

In the relapsed or refractory setting, multiple agents targeting VEGFR, PDGFR, mechanistic target of rapamycin (mTOR), and IGFR, among others, are being evaluated for pediatric sarcomas (80, 81). While multi-agent chemotherapy regimens are standard for rhabdomyosarcoma (RMS) and Ewing sarcoma, targeted therapies are increasingly being evaluated for recurrent or refractory disease. For example, a phase 1/2 trial conducted by the National Cancer Institute is evaluating the IGF-1R antibody ganitumab in combination with the Src family kinase inhibitor dasatinib in patients with embryonal or alveolar RMS refractory to other standard treatments [NCT03041701]. For patients with relapsed or refractory Ewing sarcoma, a prior phase 2 trial demonstrated partial response or stable disease following treatment with ganitumab in 55% of patients (79), and a phase 2 trial is currently being conducted to evaluate ganitumab in combination with the cyclin-dependent kinase (CDK) 4/6 inhibitor palbociclib [NCT04129151].

Desmoplastic small round cell tumor (DSRCT), a rare and aggressive STS that is characterized by translocation between EWSR1 and WT1, is typically treated with intensive multimodal therapy including alkylator-based chemotherapy, cytoreductive surgery with or without hyperthermic intraperitoneal chemotherapy (HIPEC), and whole abdominopelvic radiotherapy (82). However, survival outcomes remain dismal (5-year OS ~25%) (82), and novel therapeutic approaches are critically needed. Currently, targeted systemic therapies are usually considered at progression after first- or second-line chemotherapy, and data are limited to small case series or trials of Ewing sarcoma that include DSRCT (83). Pazopanib is one of the agents with more clinical experience that has demonstrated clinical activity in DSRCT, with partial response observed in a small subset of patients and at least stable disease observed in the majority of patients in the largest study of 22 patients with heavily pre-treated DSRCT (76, 84). Other reports have shown stable response to mTOR inhibitors and other PDGFR and VEGFR inhibitors, and a few ongoing studies are evaluating therapies targeting these and other pathways (83).

Advances in molecular and genetic tumor evaluation have allowed identification of a small subset of pediatric solid tumors that harbor targetable NTRK gene fusions and BRAF alterations (introduced above) (16, 85). A phase 1 study of the TRK kinase inhibitor larotrectinib for pediatric solid tumors harboring NTRK gene fusions demonstrated an ORR of 93% with predominantly grade 1 adverse events. Tumors included infantile fibrosarcoma, other STS, and papillary thyroid cancer (30, 86). Five patients on this phase 1 study were treated preoperatively with larotrectinib for locally advanced sarcomas, and all had radiographic partial response. Three of the five patients had R0 resections and complete or near-complete pathological responses (87). Thus, robust responses to these agents have led to their incorporation primarily for recurrent, refractory, or metastatic disease but may also be considered earlier in the course of treatment and, in rare cases, may provide an alternative to pre- or post-operative radiotherapy for management of pediatric sarcomas.

The incorporation of hypofractionated radiotherapy for local control in advanced disease settings is evolving. Stereotactic body radiotherapy (SBRT) is increasingly being utilized and studied for oligometastatic and recurrent disease, as a more convenient treatment that can minimize interruption of systemic therapy, and with possibly less toxicity than conventional radiotherapy. For sarcomas, which are typically more radioresistant, SBRT may also offer increased local control efficacy. However, the relevance and success of SBRT, which delivers high biologically effective doses to focal areas of disease, relies on improvements in micrometastatic disease control with systemic therapy. Thus, SBRT may become increasingly relevant with effective targeted systemic therapies. Several retrospective and early phase prospective studies (summarized in **Table 3**) have evaluated SBRT for metastatic and recurrent sarcomas (88–91). These have generally shown good local control outcomes, but increased toxicity when given with concurrent systemic therapy or in the re-irradiation setting. The prospective phase II study by Elledge et al. importantly suggested that survival outcomes may be improved with consolidation of all known metastatic sites with SBRT (90), consistent with data from the EURO-EWING trial indicating improved EFS with local therapy to primary and metastatic sites (92). Current COG trial AEWS1221 is evaluating SBRT for treatment of osseous metastatic sites, to a dose of 40 Gy in 5 fractions [NCT02306161]. Additional data are still needed to evaluate the safety of SBRT with newer targeted systemic therapies.

**Table 3:** Studies of stereotactic body radiotherapy for pediatric sarcomas.

Study (Treatment years)	Number of patients	Patient population	Radiotherapy site and dose	Systemic therapy	Local control and survival	Toxicity
Brown et al. (88) (2008-2012)	N=14 (27 lesions)	Retrospective study of pediatric and adult patients with metastatic or recurrent ES or osteosarcoma	Sites: Bone and lung/mediastinal Dose: Median 40 Gy in 5 fractions (range, 16-60 Gy in 1-10 fractions)	Concurrent chemotherapy in 50% of patients (n=4 with I/E, n=2 with VCR, topotecan, CP, n=1 with GEM/docetaxel, n=1 with CP/topotecan)	2-year LC: 85%	No grade ≥3 acute toxicity N=3 grade ≥2 late toxicity: sacral plexopathy (re-RT), myonecrosis (concurrent GEM), pathologic fracture/AVN
Liu et al. (89) (2017-2018)	N=5 (8 lesions)	Phase I/II study of patients ≤21 years with lung metastases from sarcoma	Sites: Lung metastases Dose: 30 Gy in 3 fractions	Concurrent systemic therapy in 1 patient (nivolumab)	2-year LC: 60%	No grade ≥3 adverse events N=1 grade ≥2 pneumonitis (concurrent nivolumab)
Elledge et al. (90) (2014-2018)	N=14 (37 lesions)	Phase II study of patients age 4-25 years with unresected, osseous metastatic non-RMS sarcomas of soft tissue and bone	Sites: Osseous metastases (spine, extremity, pelvis, skull) Dose: 40 Gy in 5 fractions	No systemic therapy within 2 weeks before or after SBRT	2-year LC: 89% (patient), 95% (lesion) Median PFS: 6 months Median OS: 24 months	N=2 grade ≥3 toxicity: esophagitis, osteoporosis/necrosis
Parsai et al. (91) (2014-2019)	N=31 (88 lesions)	Retrospective study of patients age 4-29 with recurrent or metastatic sarcomas	Sites: Osseous, pulmonary, soft tissue, hepatic Dose: Median 30 Gy in 5 fractions (range, 16-60 Gy in 1-5 fractions)	Concurrent systemic therapy with treatment of 56% of lesions (multiple agents)	1-year LC: 83%	No grade ≥3 acute toxicity N=2 grade ≥2 late toxicity: radiation enteritis (re-RT and concurrent I/E/CARBO), pain (concurrent Ra-223/sorafenib) N=2 radiation recall: dermatitis (I, mesna), myositis (paclitaxel, GEM, BEV)

AVN, avascular necrosis; BEV, bevacizumab; CARBO, carboplatin; CP, cyclophosphamide; E, etoposide; ES, Ewing sarcoma; GEM, gemcitabine; Gy, Gray; I, ifosfamide; LC, local control; OS, overall survival; PFS, progression-free survival; re-RT, re-irradiation; RMS, rhabdomyosarcoma; SBRT, stereotactic body radiotherapy; VCR, vincristine.

### Role of Immunotherapy in Management of Pediatric Cancers

While immunotherapy has revolutionized the treatment of several adult cancers, its role in pediatric malignancies has thus far been limited, in large part due to how most pediatric cancers arise: typically from embryonal cells through transcriptional abnormalities, chromosomal rearrangements, and copy number variants, as opposed to accumulation of genetic mutations in epithelial cells (93, 94). Thus, most pediatric tumors have low mutational burden and limited neoantigen expression and are non- or weakly immunogenic, with the rare exception of cancers arising from mismatch repair deficiencies (94, 95). However, a few immunotherapies have been FDA-approved for treatment of pediatric cancers. Blinatumomab, a bispecific antibody targeting the B lymphocyte antigen CD19, and tisagenlecleucel, a chimeric antigen receptor (CAR)-T cell therapy targeting CD19, are approved for treatment of relapsed/refractory B-cell acute lymphoblastic leukemia (93, 96, 97). Dinutuximab is an antibody specific for disialoganglioside (GD2), a glycolipid antigen highly expressed on the surface of neuroblastoma and other embryonal tumors. The Fc portion of anti-GD2 antibodies engages receptors on monocytes, macrophages, neutrophils, and natural killer cells, which then triggers antibody-dependent cell-mediated cytotoxicity (ADCC) and complement-dependent cytotoxicity (93, 98). Based on promising initial phase I data of dinutuximab alone and in combination with granulocyte-macrophage colony stimulating factor (GM-CSF) and interleukin-2 (IL-2) to enhance ADCC (99–101), the COG conducted the randomized phase 3 study ANBL0032 to evaluate the addition of dinutuximab with GM-CSF and IL-2 to standard isotretinoin post-consolidation therapy for high-risk neuroblastoma patients. The study was stopped early due to the superiority of the dinutuximab arm at 2 years, with significant improvements in EFS (66 ± 5% *vs*. 46 ± 5%, *P*=0.01) and OS (86 ± 4% *vs*. 75 ± 5%, *P*=0.02) (102). Thus, dinutuximab is FDA-approved for treatment of high-risk neuroblastoma patients with response to frontline multi-modal therapy (including consolidative radiotherapy) and is a standard component of post-consolidation therapy on the current COG trial ANBL1531 [NCT03126916] (93). Finally, immune checkpoint inhibitors, which have had significant success in the treatment of adult cancers, have not yet been widely adopted in the pediatric setting. Pembrolizumab, an antibody specific for programmed cell death protein 1 (PD-1) expressed on activated T and B lymphocytes, is approved for the treatment of refractory or relapsed Hodgkin lymphoma based on data extrapolated from adult studies (93, 103). Ipilimumab, an antibody targeting cytotoxic T lymphocyte antigen 4 (CTLA-4), is approved for treatment of unresectable or metastatic melanoma in pediatric patients ≥12 years of age (104, 105).

Ongoing clinical trials evaluating various immunotherapies (including immune checkpoint inhibitors, CAR-T cell therapies, cancer vaccines, and oncolytic virus therapies, among others) across a spectrum of pediatric cancers are summarized in Hutzen et al. (93) A handful of trials incorporate radiotherapy, either in combination with immunotherapy or as consolidative therapy after upfront systemic therapy. For patients ≥12 years of age with newly diagnosed stage III-IV classic Hodgkin lymphoma, a randomized phase 3 trial is evaluating immunotherapy (nivolumab, an anti-PD-1 antibody, *versus* brentuximab vedotin, an antibody-drug conjugate targeting CD30 on the surface of Hodgkin lymphoma cells) with standard combination chemotherapy followed by consolidative radiotherapy as clinically indicated [SWOG S1826, NCT03907488]. A few studies are investigating combinations of immunotherapy and radiotherapy for progressive or recurrent primary brain tumors. Indoximod is an inhibitor of the indoleamine 2,3-dioxygenase (IDO) pathway, which serves multiple immunomodulatory functions but ultimately results in immune tolerance to tumor antigens (106). A phase 1 trial of indoximod combined with temozolomide or radiotherapy for pediatric patients with progressive brain tumors (or with radiotherapy for patients with newly diagnosed DIPG) has completed enrollment [NCT02502708], and a phase 2 trial is now underway [NCT04049669]. Other studies are investigating intratumoral virus injection together with radiotherapy for malignant gliomas or recurrent ependymomas [NCT02457845, NCT00634231], as well as adoptive cellular therapy with radiotherapy (with or without temozolomide) for patients with brainstem gliomas [NCT03396575]. Finally, based on pre-clinical and clinical data suggesting more robust systemic immune responses to combinations of focal radiotherapy and immunotherapy (107–113), a few early studies are evaluating this combination in extracranial solid tumors and lymphomas [NCT03445858].

## Tailoring Radiotherapy With Advancements in Molecular Characterization

### Dose-Reduced Radiotherapy for Patients With Low Risk Medulloblastoma

Medulloblastoma is standardly treated with an aggressive multi-modal regimen of maximal safe resection followed by post-operative craniospinal irradiation (CSI) and multi-agent chemotherapy. However, as the median age at diagnosis is ~6 years of age and the majority of patients are long-term survivors (5-year OS ~80% for patients with standard risk disease and ~60% for patients with high risk disease), all patients experience late toxicities, including neurocognitive impairment, neuroendocrine dysfunction, impact on growth, infertility, and secondary malignancies, and strategies to decrease late effects from treatment while maintaining survival rates are constantly being evaluated (114). Patients with standard risk disease per traditional definitions (≤1.5cm^2^ residual disease, ≥3 years old, and no metastatic disease) are treated with lower dose CSI (23.4 Gy) with an involved field boost to 54 Gy total, while patients with high risk disease per traditional definitions (>1.5cm^2^ residual disease or metastatic disease present) are treated with higher dose CSI (36 Gy) with a posterior fossa boost to 54 Gy total and metastatic site boost to 45-54 Gy total. CSI has been an essential component of treatment for medulloblastoma, as cure was rare before the use of CSI, and early efforts to omit or reduce the dose of CSI resulted in worse outcomes (115, 116). Based on early studies of medulloblastoma demonstrating significant and often unacceptable neurocognitive deficits attributed to high dose radiotherapy in children under the age of 3 (1, 117, 118), radiotherapy is typically delayed for infants and young children with medulloblastoma until age 3 or older. Surgical resection is usually followed by adjuvant chemotherapy, delaying radiotherapy until progression (the “acceptable” age for proceeding with CSI varies across studies, from 18 months to 6 years) (119–121). The COG trial ACNS0334 [NCT00336024] is evaluating two high-dose chemotherapy regimens followed by peripheral blood stem cell rescue for infants up to age 2 with high-risk medulloblastoma or CNS embryonal tumors, and preliminary results suggest that while focal radiotherapy may be reasonable upfront for select patients, omission of CSI upfront does not appear to compromise survival (122).

Newer radiotherapy techniques, including IMRT and proton therapy, as well as reduction in the boost margin, have resulted in steadily lower doses to normal tissues without compromising disease control (123–125). Specifically for proton therapy, dosimetric studies indicate reduction of dose to anterior organs, including heart, gastrointestinal tract, lungs, kidneys, and thyroid, with proton CSI (126), and evaluation of long-term toxicity of proton therapy for medulloblastoma suggests decreased cardiac, pulmonary, and gastrointestinal toxicity compared to photon-based treatments (127). While neurocognitive impairment will always occur with CSI regardless of treatment modality, especially with younger age at the time of treatment (128), a recent study suggests that better intellectual outcomes may still be achieved with proton *versus* photon radiotherapy for medulloblastoma based on the boost treatment (129). Thus, even with standard-dose radiotherapy for medulloblastoma, advancements in radiotherapy techniques are resulting in improvements in the late toxicity profile.

**Figure 3:**
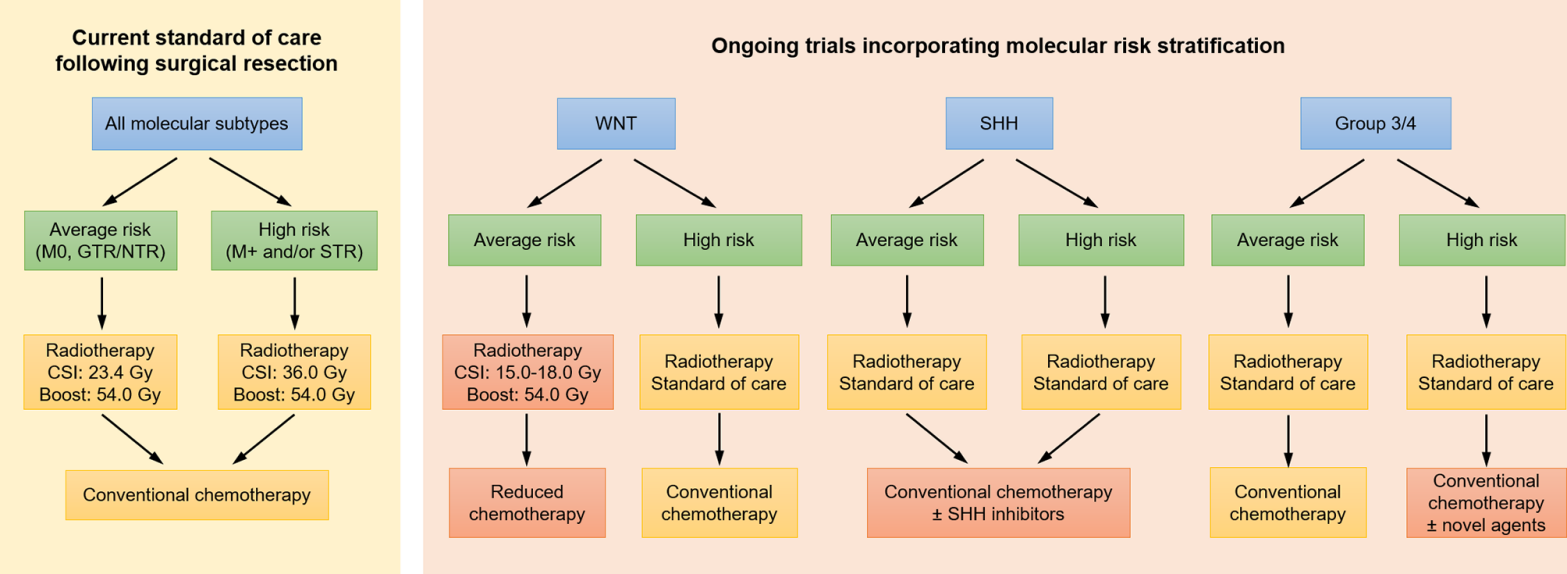
Current treatment paradigms for medulloblastoma, as well as approaches under investigation in clinical trials incorporating molecular risk stratification. Investigational approaches are indicated in red. CSI, craniospinal irradiation; GTR, gross total resection; NTR, near total resection; SHH, sonic hedgehog; STR, subtotal resection.

More recently, the management of medulloblastoma has been revolutionized by advancements in tumor molecular characterization, moving from previous risk definitions based on amount of residual disease, age, and presence of metastatic disease to current stratifications based on molecular subgroups: WNT, sonic hedgehog (SHH), Group 3, and Group 4. With standard treatments, the WNT subgroup is most favorable, with >90% 5-year PFS, followed by intermediate outcomes in the SHH and Group 4 subgroups (5-year PFS of 70-80%), and poor outcomes for Group 3 (5-year PFS of 50-60%) (8, 114, 130). Thus, current studies are evaluating whether patients in low risk subgroups may be eligible for de-intensified treatment regimens, whether avoiding radiotherapy altogether or reducing the dose or volume of radiotherapy (**Figure 3**) (7, 130). COG study ACNS1422 [NCT02724579] is evaluating whether both chemotherapy intensity and CSI dose (18 Gy) can be reduced in patients with average risk WNT-driven tumors who have positive β-catenin and presence of *CTNNB1* [exon 3] mutation and without large cell/anaplastic medulloblastoma or *MYC/MYCN* amplification. SJMB12 [NCT01878617] is evaluating a reduced CSI dose of 15 Gy in the same population. However, a pilot study omitting CSI entirely for WNT-driven medulloblastoma has closed due to inferior outcomes [NCT02212574]. In Europe, the ongoing International Society of Paediatric Oncology (SIOP) PNET-5 study is investigating the possibility to deliver, within a combined modality approach, a reduced CSI dose of 18 Gy to a selected subgroup of children with a low-risk biological profile [NCT02066220]. At the same time, SJMB12 is investigating intensified treatment regimens for patients in higher risk subgroups, including the addition of gemcitabine and pemetrexed for those with high risk Group 3 or Group 4 medulloblastoma and targeted SHH inhibitor therapy for those with SHH-medulloblastoma (**Figure 3**).

### Risk-Adapted Radiotherapy for Patients With Rhabdomyosarcoma

Rhabdomyosarcoma is standardly treated with a combined modality regimen of surgery (if resectable), multi-agent chemotherapy, and radiotherapy. Use and dose of radiotherapy for rhabdomyosarcoma is typically based on clinical group, FOXO1 fusion status, and site (primary/metastatic). Patients with clinical group I, FOXO1 negative or indeterminate tumors do not receive radiotherapy, while all others receive radiotherapy with dose based on the factors above. Given the young age of many of these patients and significant risk of late toxicity from radiotherapy (131–134), there is always a question of whether radiotherapy can be safely omitted or reduced and thereby minimize treatment-related toxicity for appropriately selected patients. An analysis of ARST0331 and ARST0531 suggests worse local control and survival outcomes when “individualized local therapy” (typically omission or delay of radiotherapy) as opposed to protocol-specified radiotherapy is given to infants with rhabdomyosarcoma (135). Thus, attempting to select for more favorable risk patients, the current protocol ARST1431 [NCT02567435] permits deviations for patients ≤2 years of age only if they are FOXO1 fusion negative. Histologic and radiographic response to initial chemotherapy is another measure that has been used to guide radiotherapy usage and dose (used in D9602/D9803 and ARST0331/ARST0531, as well as in ARST1431). Second-look procedures after initial chemotherapy largely correlate with clinical/radiographic complete response; however, ~40% of patients without clinical/radiographic complete response have no viable tumor histologically, and thus post-chemotherapy biopsies/DPE may be helpful for selecting patients for radiotherapy dose reduction (136). On the other end, ARST1431 is evaluating higher doses of radiotherapy in patients at greater risk of local failure by increasing the boost dose to 59.4 Gy total for tumors >5cm at diagnosis.

Future studies will need to incorporate our evolving understanding of molecular and genetic features of rhabdomyosarcoma that are associated with favorable or adverse outcomes, such that patients can be appropriately selected for potential treatment de-escalation or escalation. For instance, recent histological and molecular analysis of infant rhabdomyosarcoma suggests favorable prognosis of the spindle cell subtype associated with alterations in VGLL2, NTRK, and BRAF, and potential consideration of de-intensified treatment for this subset of patients (137). Conversely, MYOD1-mutant spindle cell and sclerosing rhabdomyosarcoma is associated with an aggressive clinical course and poor outcomes (138, 139) and, together with tumors with anaplasia and TP53 mutation, should be excluded from consideration of de-escalated therapy and perhaps considered for augmented therapy.

## Conclusion

Radiotherapy has remained an integral component in the treatment of pediatric cancers over several decades. However, its role has continued to evolve with the introduction of chemotherapy regimens and now molecularly targeted therapies in an era of rapid advances in precision medicine. In particular, MEK1/2, BRAF, and TRK inhibitors have demonstrated significant promise in pediatric gliomas and extracranial solid tumors harboring these alterations and warrant further investigation in larger trials, as well as clinical consideration when these alterations are present. Developments in molecular diagnostics and targeted systemic therapies are providing opportunities for potentially more effective and specific but less toxic therapies, critical for treatment of pediatric patients. At the same time, advances in radiotherapy techniques are improving the precision and conformality of local therapy. Together, these developments are leading to novel synergistic combinations of radiotherapy and systemic therapy, as well as potential avenues to select patients for treatment de-escalation, leading to more tailored treatments with improved therapeutic ratio for pediatric cancer patients.

## Author Contributions

CS and ST contributed to conception of the manuscript. CS wrote the first draft of the manuscript. All authors contributed to the article and approved the submitted version.

## Conflict of Interest

The authors declare that the research was conducted in the absence of any commercial or financial relationships that could be construed as a potential conflict of interest.

## Publisher’s Note

All claims expressed in this article are solely those of the authors and do not necessarily represent those of their affiliated organizations, or those of the publisher, the editors and the reviewers. Any product that may be evaluated in this article, or claim that may be made by its manufacturer, is not guaranteed or endorsed by the publisher.
